# Feature Extraction Methods for Underwater Acoustic Target Recognition of Divers

**DOI:** 10.3390/s24134412

**Published:** 2024-07-08

**Authors:** Yuchen Sun, Weiyi Chen, Changgeng Shuai, Zhiqiang Zhang, Pingbo Wang, Guo Cheng, Wenjing Yu

**Affiliations:** 1 Institute of Noise and Vibration, Naval University of Engineering, Wuhan 430033, China; such027@163.com (Y.S.);; 2 National Key Laboratory on Ship Vibration and Noise, Wuhan 430033, China; 3 Academy of Weapony Engineering, Naval University of Engineering, Wuhan 430033, China; 4 Academy of Electronic Engineering, Naval University of Engineering, Wuhan 430033, China

**Keywords:** underwater acoustics, target recognition, feature extraction, support vector machine, diver

## Abstract

The extraction of typical features of underwater target signals and excellent recognition algorithms are the keys to achieving underwater acoustic target recognition of divers. This paper proposes a feature extraction method for diver signals: frequency−domain multi−sub−band energy (FMSE), aiming to achieve accurate recognition of diver underwater acoustic targets by passive sonar. The impact of the presence or absence of targets, different numbers of targets, different signal−to−noise ratios, and different detection distances on this method was studied based on experimental data under different conditions, such as water pools and lakes. It was found that the FMSE method has the best robustness and performance compared with two other signal feature extraction methods: mel frequency cepstral coefficient filtering and gammatone frequency cepstral coefficient filtering. Combined with the commonly used recognition algorithm of support vector machines, the FMSE method can achieve a comprehensive recognition accuracy of over 94% for frogman underwater acoustic targets. This indicates that the FMSE method is suitable for underwater acoustic recognition of diver targets.

## 1. Introduction

The underwater acoustic recognition technology for small underwater targets such as divers has always been a challenge worldwide. The main reason for this is not only the wet end (the underwater part of the sonar system, e.g., hydrophones) of sonar equipment with high technical requirements but also the dry end (the onshore part of the sonar system, e.g., computers) of the extraction of signal features and target recognition algorithms. At present, feature extraction and recognition algorithms are mainly used in the air, e.g., mechanical fault diagnosis, geological disaster prediction, and traffic signal recognition [1,2,3]. Little research has been conducted on feature extraction and recognition algorithms for underwater signal processing, which started late and has made slow progress, limiting the recognition effect on underwater acoustic targets such as divers [4].

The extraction of underwater acoustic signal features from divers is a prerequisite for achieving underwater acoustic target recognition. To achieve a higher probability of target recognition under the same conditions, it is particularly crucial to know whether the extracted target signal features are typical, in addition to having excellent recognition algorithms.

At present, the extracted target features of underwater acoustic signals mainly include mathematical features, e.g., waveform features, wavelet features, and auditory features. In the field of time−domain waveform analysis, Meng has developed mathematical features involving peak−to−peak amplitude and zero intersection features [5], but the effect of feature extraction is not ideal in complex backgrounds. The feature extraction method based on time/frequency analysis is more suitable for non−stationary fields and has been widely used. These feature extraction methods mainly include the Hilbert Huang transform [6], the wavelet, and other transformation methods. Sadjadi and Yao solved the feature extraction method based on wavelet packets [7]. Wang weighted and fused wavelet features with principal component analysis to obtain sound signal features to recognize underwater targets [8], but a lack of prior experience made it difficult for researchers to rank the components of the wavelet decomposition.

Due to the non−static and time−varying characteristics of underwater signals, traditional feature extraction methods are difficult to apply directly in the field of underwater target recognition. The non−equal frequency filtering and other functions of the human auditory system have been found to effectively distinguish between background interference and target signals [9]. With the non−equal frequency band−pass filtering characteristics of the human ear, the mel frequency cepstral coefficient (MFCC) feature extraction method has been developed, which is gradually gaining popularity in underwater target feature extraction [8]. Lim et al. are one of the earliest groups to apply the MFCC method to underwater target recognition and achieve ideal results [10]. Bae et al. applied MFCC underwater acoustic signal features to the classification method of a neural network and achieved good results [10].

The underwater feature extraction method using the gammatone frequency cepstral coefficient (GFCC) is studied later. This method has a better non−equal frequency filtering effect [11] and higher robustness, which can make GFCC’s voiceprint feature extraction of underwater targets more typical in cluttered backgrounds [12]. In 2014, Zeng and Wang combined GFCC features with a Hilbert Huang transform for underwater target recognition, verifying that this method has better classification performance than MFCC features. Since then, GFCC features have become an important and popular research topic [13].

Nevertheless, limited research worldwide has been conducted on the feature extraction methods of underwater acoustic signals for divers, which leads to limitations in the large−scale application of existing sonar fields. The time−frequency domain characteristics of underwater acoustic signals from divers are highly intuitive and can distinguish the presence or absence of underwater diver targets effectively with the help of manual assistance [14]. However, the typical components of its signal features are difficult to extract mathematically, and the signal feature data is complex, with high−dimension information. Using these high−dimension signal features to achieve automatic discrimination of underwater targets requires a large amount of computation and has low accuracy [15]. Therefore, it is necessary to develop new and high−quality feature extraction methods to reduce the data dimension of signal features, highlight the features of target signals, and achieve efficient and accurate recognition of underwater diver targets.

To achieve better feature extraction results, this work employed three feature extraction methods: frequency−domain multi−sub−band energy (FMSE), mel frequency cepstral coefficient filtering (MFCC), and gammatone frequency cepstral coefficient filtering (GFCC) for underwater acoustic target recognition of divers, according to the multi−frequency resolution principle of the human cochlear basement membrane [16]. The SVM recognition algorithm that was often used as classifiers for target recognition for faster learning speed, higher classification generalization ability, and smaller recognition errors compared to methods such as neural networks [17] was combined for comprehensive comparison to test the performance of these three feature extraction methods, which is expected to provide a feature extraction method to improve the effectiveness of underwater diver target recognition.

## 2. Method for Extracting Underwater Acoustic Signal Features of Divers

To achieve efficient and accurate recognition of underwater acoustic signals from divers, it was necessary to map the high−dimension features of the diver’s signal to a low−dimension space through feature extraction methods to obtain low−dimension typical features that can represent the diver targets. To this end, this work introduced three feature extraction methods: FMSE, MFCC, and GFCC, into the field of underwater acoustic recognition of divers.

### 2.1. FMSE Feature Extraction Method

The energy features of signal sub−bands were divided into time−domain multi−sub−band energy features and FMSE features. The algorithm of time−domain multi−sub−band energy features was used to divide the signal time domain into multiple sub−bands, calculate the energy of each sub−band, and then convert this data into the frequency domain through a Fourier transform. According to the frequency−domain envelope, it was determined whether there was a prominent signal consistent with the breathing frequency of the diver, which could be used to achieve the discrimination of the diver. However, this method may cause secondary frequency interference in the actual process; when the signal−to−noise ratio (SNR) was low, this method could not effectively highlight the breathing cycle of the diver’s signal [18]. The FMSE algorithm was first used to convert the time−domain signal into a frequency−domain signal, and then perform band−pass filtering and sub−band division to calculate the energy of each sub−band signal. During the calculation process, FMSE can effectively improve the SNR without secondary frequency interference.

The distribution of underwater acoustic signal energy in various frequency bands was different based on whether a diver was present or not. When a diver was present underwater, the energy distribution of the signal time−frequency spectrum in the frequency domain showed a certain regularity, which was the frequency−domain energy features of the diver’s signal. By calculating the energy distribution of the diver’s signal in each sub−band of the prominent frequency band (2–8 kHz) [19], a unique frequency−domain multi−sub−band energy sequence of the diver’s signal was obtained, which can be considered as a signal feature vector for recognizing divers.

**Figure 1:**
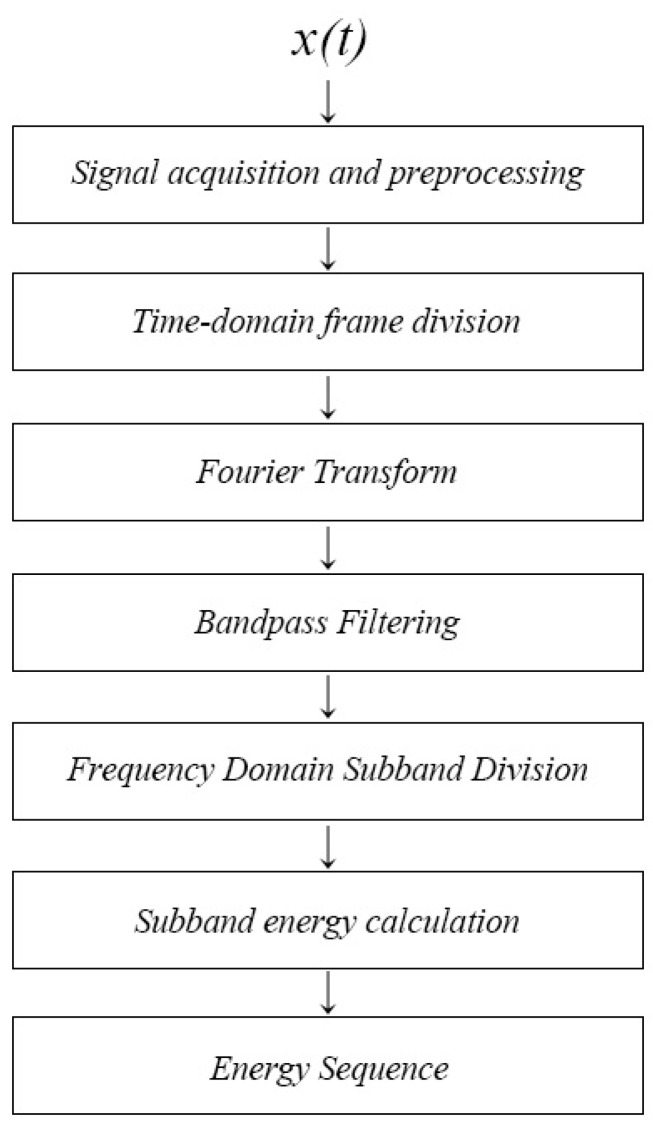
Calculation flow chart of the frequency−domain multi−sub−band energy feature extraction method.

As shown in Figure 1, the flow of the FMSE calculation method in this paper was as follows:

(1) Signal acquisition and preprocessing: obtain the time−domain signal x(t) collected by the hydrophone as the signal to be processed, ignore the multipath effects and propagation losses, and perform x(t) signal preprocessing, including de−trending, pre−emphasis, and noise reduction.

(2) Time−domain frame division: take x(t) as a continuous signal of approximately 10 s in length, divide it into frames, and apply a window function; take *N* = 1024 sampling points as one frame and perform frame window sliding (Hamming window, frameshift to *N*/2, overlap rate 50%), and the *k*th frame signal was $x\left[k\right]$ (1 × *N* dimension):

(1) $$x\left[k\right]=\int_{(k-1)T}^{kT}x(t)\delta (t-\tau )d\tau ,$$

where *T* was the duration of each frame and δ(t) was the unit impulse signal.

(3) Fourier transform: each frame was regarded as a second−order stationary signal and a discrete Fourier transform was performed on the signal of each frame. The signal of the *k*th frame after transformation was $X_{k}(j\omega )$ (1 × *N* dimension):

(2) $$X_{k}(j\omega )=\sum_{n=0}^{N-1}x\left[k\right]e^{-j\omega k}.$$

(4) Bandpass filtering: bandpass filtering was performed by the upper and lower limits of the prominent frequency band of the diver’s signal for $X_{k}(j\omega )$.

(5) Frequency−domain sub−band division: within the prominent frequency band, the filtered signal was divided into non−overlapping equal frequency−domains, resulting in *m* individual frequency sub−bands in each protruding frequency band where *m* is a positive integer. Based on past research experience [20], the filtered signal was generally divided into 5–10 sub−bands. If the value of *m* was too large, it would increase the computational burden, and if the value of *m* was too small, the signal details would not be fully revealed. In this paper, the number of sub−bands was set to *m* = 8.

(6) Sub−band energy calculation: the signal within each sub−band was considered to be linear and stationary, and the energy of the signal within each sub−band was calculated, which was the desired sub−band energy; the signal of the *i*th (*i* ≤ *m*) sub−band was $X_{k}(f_{i})$, and its energy was $E_{i}$. The arrangement and combination of the sub−band energy $\vec{E}$ was the sequence feature of the signal’s sub−band energy.

The m−dimension sub−band energy sequence within each prominent frequency band was:

(3) $$\vec{E}={\left[E_{1},E_{2},E_{3},\ldots ,E_{m}\right]}^{T}\;m=8.$$

Among them, the energy of the *i*th sub−band $E_{i}$ was:

(4) $$E_{i}={\left\|X_{k}(f_{i})\right\|}^{2}\;i=1,2\ldots m,$$

where $\left\|*\right\|$ represents the 2−norm of a vector.

### 2.2. MFCC Feature Extraction Method

The MFCC feature extraction algorithm was used to preprocess the time−domain signal x(t) collected by the hydrophone; this was divided into frames and a window function was applied followed by a Fourier transform; then, a set of triangular filters with mel scales was applied to the energy spectrum, followed by a logarithmic operation to extract signal features.

**Figure 2:**
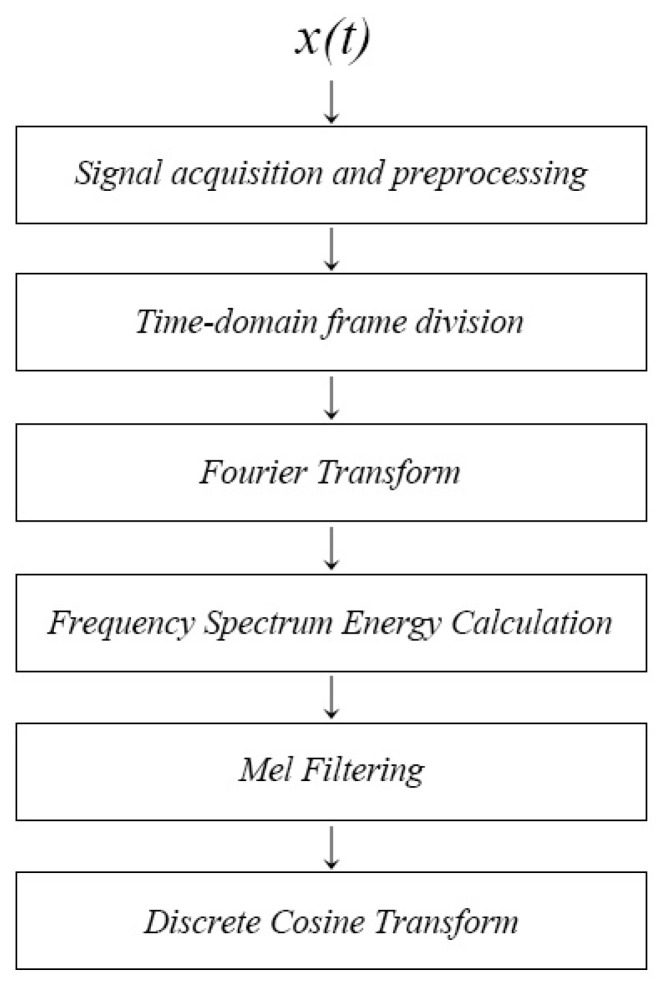
Calculation flow chart of mel frequency cepstral coefficient feature extraction method.

As shown in Figure 2, the flow of the MFCC calculation method in this paper was as follows [21]:

(1) Signal acquisition and preprocessing: obtain the time−domain signal x(t) collected by the hydrophone as the signal to be processed, ignore the effects of multipath effects and propagation losses, and perform signal x(t) preprocessing (de−trending, pre−emphasis, and noise reduction).

(2) Time−domain frame division: take x(t) a continuous signal of approximately 10 s in length, divide it into frames, and apply a window function; take *N* = 4096 sampling points as one frame, and perform frame window sliding (Hamming window, frameshift to *N*/2, overlap rate 50%), and the number *k* frame signal $x\left[k\right]$ was:

(5) $$x\left[k\right]=\int_{(k-1)T}^{kT}x(t)\delta (t-\tau )d\tau ,$$

where *T* was the duration of each frame and δ(t) was the unit impulse signal.

(3) Fourier transform: Each frame was regarded as a second−order stationary signal, and the discrete Fourier transform was performed on the signal of each frame. The *i*th line spectrum of the *k*th frame signal after transformation was $X_{k}(i)$:

(6) $$X_{k}(i)=\sum_{n=0}^{N-1}x\left[k\right]e^{-j\omega k}.$$

(4) Frequency spectrum energy calculation: calculate the spectral energy of each frame of Fourier−transformed signal data.

(7) $$E_{k}(i)={\left|X_{k}(i)\right|}^{2}.$$

(5) Mel filtering: filter the spectral energy of each frame through a mel filter bank and calculate the energy in the filter.

**Figure 3:**
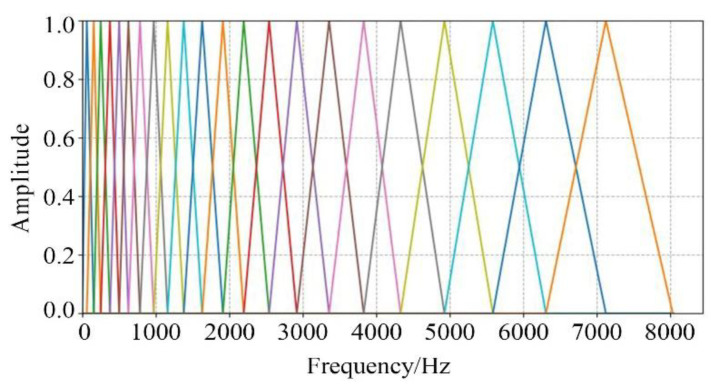
Amplitude−frequency response curves of mel triangular filter bank.

The transfer function of the mel filter bank was:

(8) $$H_{m}(k)=\left\{\begin{array}{cc} 0 & k<f(m-1)\mathrm{or}\;k>f(m+1)\\ \frac{2\left(k-f(m-1)\right)}{\left(f(m+1)-f(m-1)\right)\left(f(m)-f(m-1)\right)} & f(m-1)\leq k\leq f(m)\\ \frac{2\left(f(m+1)-k\right)}{\left(f(m+1)-f(m-1)\right)\left(f(m)-f(m-1)\right)} & f(m)\leq k\leq f(m+1), \end{array}\right.$$

where $H_{m}(k)$ represented the *m*th band−pass filter within the frequency spectrum range of the signal, and 0≤m<M (*M* is the number of filters, which was taken as *M* = 24 in this paper), and $\sum_{m=0}^{M-1}H_{m}(k)=1$, f(m) was the center frequency of each filter. The amplitude−frequency response curves of the mel triangular filter bank are shown in Figure 3.

Next, the logarithm of the energy output by each filter bank was calculated using Equation (9):

(9) $$S(m)=\ln\left(\sum_{k=0}^{N-1}{\left|X_{k}(i)\right|}^{2}\right)H_{m}(k).$$

(6) Discrete cosine transform: perform a discrete cosine transform on the logarithmic output data to obtain the MFCC coefficient, as follows:

(10) $$mfcc(n)=\sum_{k=0}^{N-1}S(m)\cos\left(\frac{\pi n(m-0.5)}{M}\right),n=1,2,\ldots ,L,$$

where *L* represented the order of the MFCC coefficient.

In this case, mfcc(n) only reflected the static characteristic coefficient of the signal. To further extract the dynamic features of the signal, the MFCC features can be further differentiated to obtain first− (Δmfcc(n)) and second− (ΔΔmfcc(n)) order differential coefficient using Equations (11) and (12), respectively, as follows:

(11) $$\Delta mfcc(n)=\frac{\sum_{w=1}^{W}w\left(mfcc(n+w)-mfcc(n-w)\right)}{2\sum_{w=1}^{W}w^{2}},$$

(12) $$\Delta \Delta mfcc(n)=\frac{\sum_{w=1}^{W}w\left(\Delta mfcc(n+w)-\Delta mfcc(n-w)\right)}{2\sum_{w=1}^{W}w^{2}}.$$

In the actual target recognition process, the first and last dimensions of the MFCC coefficient were generally used because of their strong discriminative performance. The 12 dimensions of mfcc(n) and Δmfcc(n) were respectively extracted to form 24−dimension feature vectors in this paper for ΔΔmfcc(n) having weak discriminative performance on target features [22].

### 2.3. GFCC Feature Extraction Method

The GFCC feature extraction algorithm was used to preprocess the time−domain signal x(t) collected by a hydrophone, divide it into frames, and apply a window function, then apply a set of gammatone filter banks and perform a cubic nonlinear calculation, and finally, extract signal features through a discrete cosine transform.

**Figure 4:**
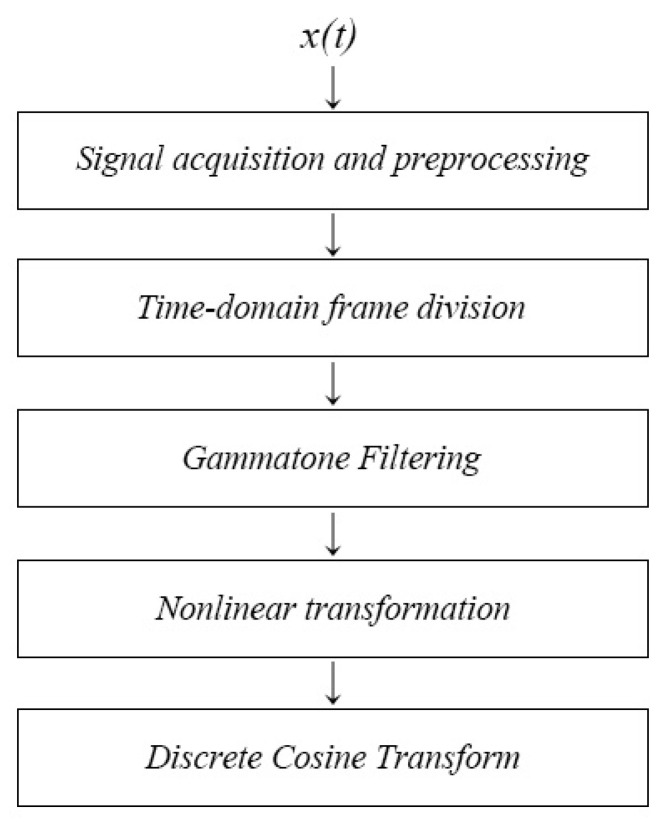
Calculation flow chart of gammatone frequency cepstral coefficient feature extraction method.

As shown in Figure 4, the flow of the GFCC calculation method in this paper (Figure 4) used the following process:

(1) Signal acquisition and preprocessing: obtain the time−domain signal x(t) collected by the hydrophone as the signal to be detected, ignore the effects of multipath effects and propagation losses, and perform signal x(t) preprocessing, including de−trending, pre−emphasis, and noise reduction.

(2) Time−domain frame division: take x(t) a continuous signal of approximately 10 sec in length, divide it into frames, and apply a window function, take *N* = 1024 sampling points as one frame, and perform frame window sliding (Hamming window, frameshift to *N*/2, overlap rate 50%), and the *k*th frame signal $x\left[k\right]$ was:

(13) $$x\left[k\right]=\int_{(k-1)T}^{kT}x(t)\delta (t-\tau )d\tau ,$$

where *T* is the duration of each frame and δ(t) is the unit impulse signal.

(3) Gammatone filtering: filter the *k*th frame signal $x\left[k\right]$ through a gammatone filter bank, the number of channels of the filter was taken as *m* = 24 in this paper. The output value of each channel was the weighted sum of the signal amplitude. The output of all channels constituted an *m*−dimension feature vector $\vec{S}(k)$:

(14) $$\vec{S}(k)={\left[s_{1}(k),s_{2}(k),s_{3}(k),\ldots ,s_{m}(k)\right]}^{T}.$$

Among them, the time−domain function of the gammatone filter bank was:

(15) $$g(t)=\alpha t^{n-1}e^{-2\pi bt}\cos\left(2\pi ft+\phi \right)t\geq 0,$$

where t was time (t≥0), α was the gain of the filter amplitude (α=1 in this paper), n was the filter order (n=4 in this paper), f was the center frequency of the filter, ϕ was the signal phase (ϕ=0 in this paper, based on the human ear’s insensitivity to phase), and b was the critical frequency band of the signal, where the value of b determines the bandwidth of the corresponding filter and is usually taken as 1.019. The relationship between f and b was:

(16) b=24.7(4.37f/1000+1).

**Figure 5:**
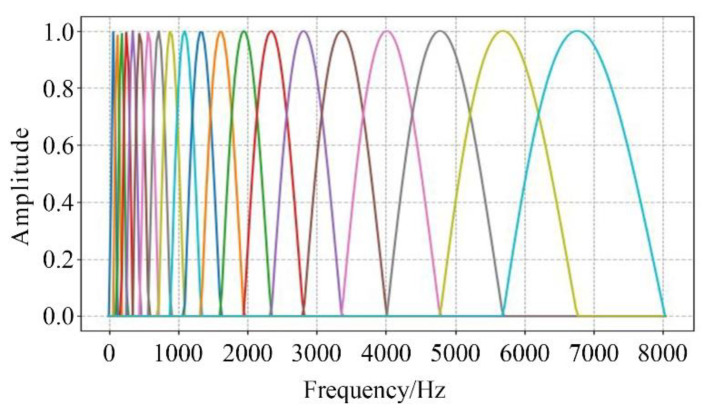
Amplitude–frequency response curves of gammatone filter bank.

The amplitude–frequency response curves of the gammatone filter bank with different center frequencies are shown in Figure 5, and the bandwidth widened as the center frequency increased.

(4) Nonlinear transformation: perform cube root extraction on *m*−dimension feature vectors $\vec{S}(k)$ to better simulate the nonlinear resolution function of the human ear.

(5) Discrete cosine transform: perform discrete cosine transform on the cubed feature vectors, which can achieve a certain reduction in dimensionality effect and remove the correlation between adjacent features; the feature parameters of the number 0 order were usually not considered recognition features.

In summary, the calculation method for n−order GFCC features was as follows:

(17) $$\vec{G}(n)={\left(\frac{2}{m}\right)}^{0.5}\sum_{i=1}^{m}\left\{s{\left(i\right)}^{1/3}\cos\left[\frac{\pi n}{2m}\left(2i-1\right)\right]\right\}.$$

## 3. Results

### 3.1. Results of FMSE Feature Extraction

According to the FMSE method introduced in this paper, the eight−dimension FMSE feature had been extracted in the frequency band of 2 to 8 kHz for underwater signals of divers under various operating conditions, including the presence or absence of divers and differences in the numbers of divers, SNRs, and detection distances, hoping to extract the typical sub−band energy distribution feature of underwater diver signals.

(1) Presence or absence of divers

**Figure 6:**
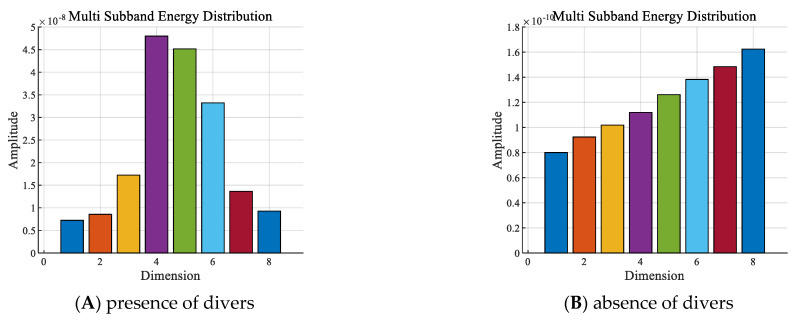
Sub−band energy sequences with the: (**A**) presence or (**B**) absence of divers.

With other conditions being equal, the sub−band energy sequences of the presence and absence of divers underwater within the 2 to 8 kHz frequency band are shown in Figure 6.

As shown in Figure 6, in the frequency band of 2 to 8 kHz:

① The amplitude of the sub−band energy with divers was significantly higher than that without divers. In the corresponding signal dimension, the sub−band energy amplitude with divers was about two orders of magnitude higher than that without divers.

② The sub−band energy increased sequentially with the dimensionality when there were no divers. When there were divers, the sub−band energy was mainly distributed in the 4th to 6th dimensions, which was very different from the features without divers.

(2) Different numbers of divers underwater.

**Figure 7:**
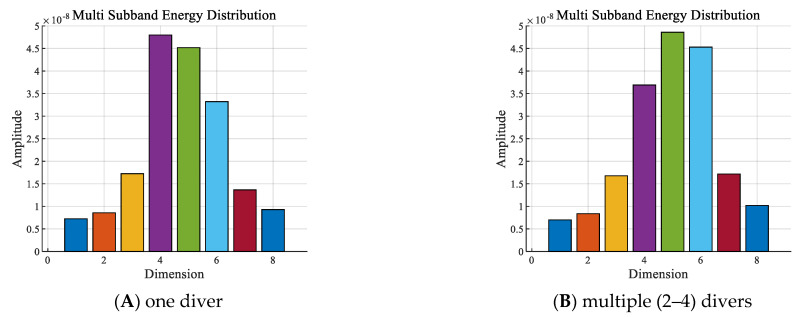
Sub−band energy sequences with the presence of: (**A**) one or (**B**) two to four divers.

With other conditions being equal, the sub−band energy sequences of the signals of one diver and multiple (2−4) divers underwater within the 2−8 kHz frequency band are shown in Figure 7.

As shown in Figure 7:

① In the 2 to 8 kHz frequency band, the sub−band energies of the signals were mainly distributed in the 4th to 6th dimensions whether with one diver or multiple divers were in the water, all of which were significantly different from the pure background noise in Figure 6B, and the amplitudes of the corresponding dimensional energy features of the two were very close.

② From the 4th to the 6th dimension features, the signal sub−band energy of one diver underwater was mainly concentrated on the 4th to 5th dimensions, while the signal sub−band energy of multiple divers underwater was mainly concentrated on the 5th to 6th dimensions. However, the amplitudes of the corresponding dimensional energy features for both were very close to the same, and the features were not clearly distinguishable.

(3) Different SNRs

**Figure 8:**
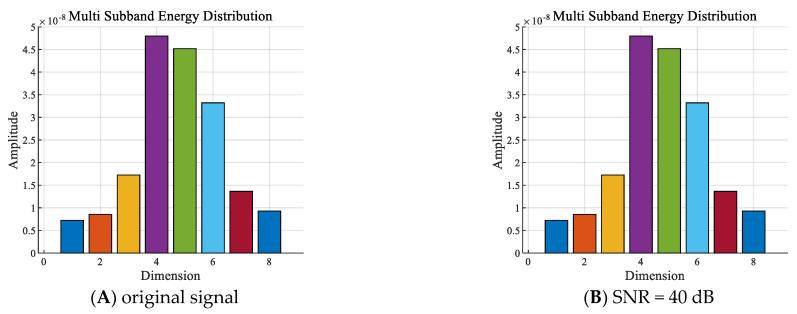

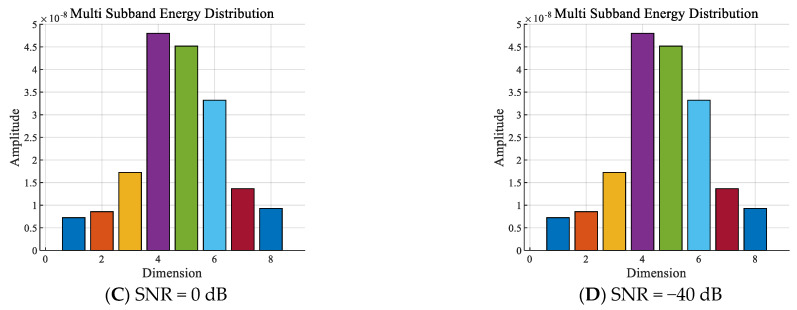
Sub−band energy sequences of one diver with: (**A**) the original signal−to−noise ratio (SNR), (**B**) 40 dB SNR, (**C**) 0 dB SNR, and (**D**) −40 dB SNR.

With other conditions being equal, the shallow sea background noise was added to the signals of one diver underwater in the 2 to 8 kHz frequency band to construct sub−band energy sequences for different SNRs, as shown in Figure 8.

Figure 8 shows that the sub−band energy distributions of the signals were basically not affected by the SNRs within the frequency band of 2 to 8 kHz.

(4) Different detection distances

**Figure 9:**
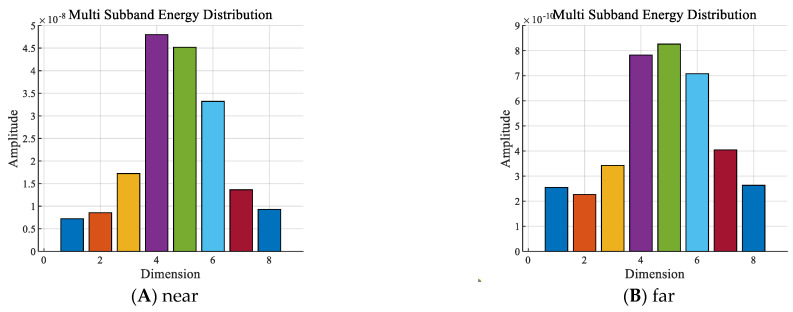
Sub−band energy sequences of one diver: (**A**) ~2 m and (**B**) ~20 m from the hydrophone.

With other conditions being equal, the sub−band energy sequences of the signals with one diver underwater are shown in Figure 9 in the frequency band of 2 to 8 kHz, near the hydrophone (about 2 m away) and far away (about 20 m away).

As shown in Figure 9, in the frequency band of 2 to 8 kHz, regardless of whether the diver was about 2 m from the hydrophone or about 20 m away, the sub−band energies of the signals were mainly distributed in the 4th to 6th dimensions, which were significantly different from the pure background noise in Figure 6B. However, the feature amplitudes of the diver signals nearby were about two orders of magnitude higher than those of the signals at about 20 m away in the corresponding dimensions.

### 3.2. Results of MFCC Feature Extraction

According to the method of MFCC feature extraction introduced in this paper, 24−dimension MFCC features were extracted in the frequency band of 2 to 8 kHz for underwater signals of divers under various operating conditions, including with/without divers, and differences in the numbers of divers, SNRs, and detection distances, hoping to extract the typical MFCC features of underwater diver signals.

(1) Presence or absence of divers

**Figure 10:**
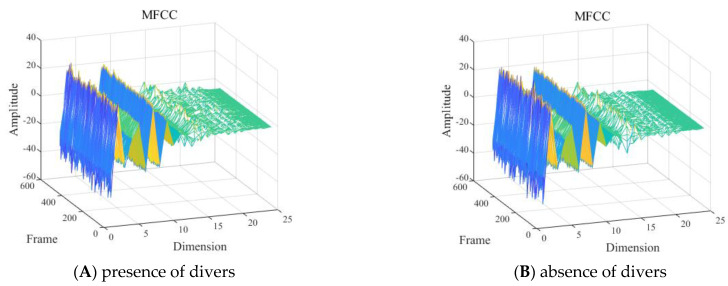
Mel frequency cepstral coefficient (MFCC) feature with the: (**A**) presence or (**B**) absence of divers.

With other conditions being equal, the MFCC feature of the presence and absence of divers underwater within the 2 to 8 kHz frequency band is shown in Figure 10.

As shown in Figure 10, it was difficult to visually determine the influence of the presence and absence of divers underwater in the frequency band of 2 to 8 kHz on the MFCC features.

(2) Different numbers of divers underwater

**Figure 11:**
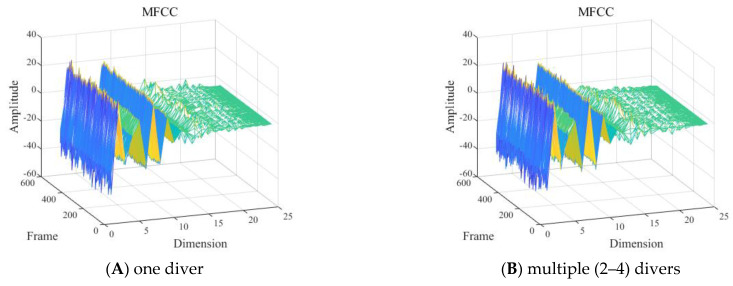
Mel frequency cepstral coefficient (MFCC) feature with the presence of: (**A**) one or (**B**) two to four divers.

With other conditions being equal, the MFCC feature of the signals of one diver and multiple (2–4) divers underwater in the 2 to 8 kHz frequency band is shown in Figure 11.

As shown in Figure 11, it was difficult to visually determine the influence of different numbers of divers of the signal in the frequency band of 2 to 8 kHz on the MFCC features.

(3) Different SNRs

**Figure 12:**
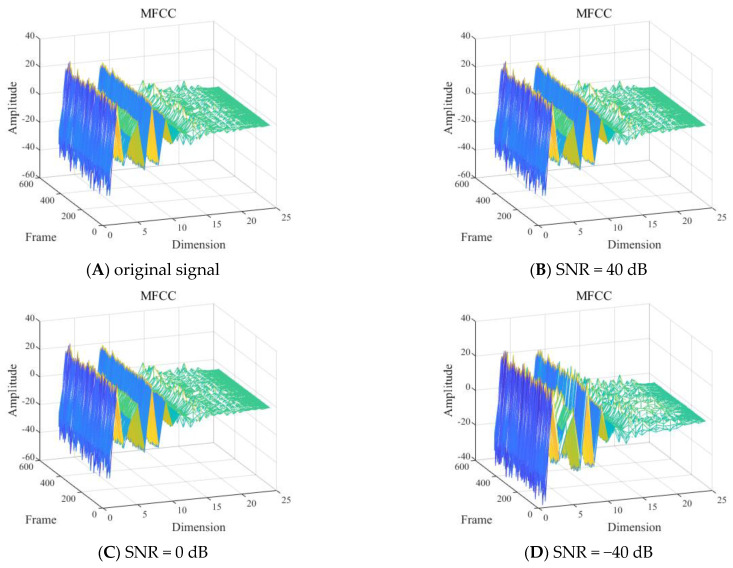
Mel frequency cepstral coefficient (MFCC) feature of one diver with: (**A**) the original signal−to−noise ratio (SNR), (**B**) 40 dB SNR, (**C**) 0 dB SNR, and (**D**) −40 dB SNR.

With other conditions being equal, the shallow sea background noise was added to the signals of one diver underwater in the 2 to 8 kHz frequency band to construct MFCC feature distributions for different SNRs, as shown in Figure 12.

As shown in Figure 12, it was difficult to visually determine the influence of different SNRs of the signal in the frequency band of 2 to 8 kHz on the MFCC features.

(4) Different detection distances

**Figure 13:**
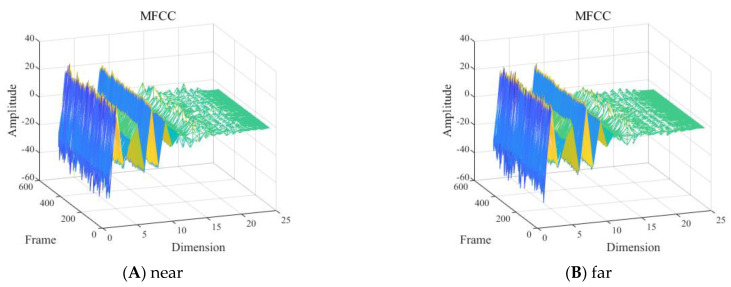
Mel frequency cepstral coefficient (MFCC) feature of one diver: (**A**) ~2 m and (**B**) ~20 m from the hydrophone.

With other conditions being equal, the MFCC features of the signals with one diver underwater in the frequency band of 2 to 8 kHz, near the hydrophone (about 2 m away) and far away (about 20 m away), are shown in Figure 13.

As shown in Figure 13, it was difficult to visually determine the influence of different detection distances of the signal in the frequency band of 2 to 8 kHz on the MFCC features.

### 3.3. Results of GFCC Feature Extraction

According to the method of GFCC feature extraction introduced in this paper, a 24−dimension GFCC feature was extracted in the frequency band of 2 to 8 kHz for underwater signals of divers under various operating conditions, including with/without divers as well as with different numbers of divers, SNRs, and detection distances, hoping to extract the typical GFCC feature of underwater diver signals.

(1) Presence or absence of divers

**Figure 14:**
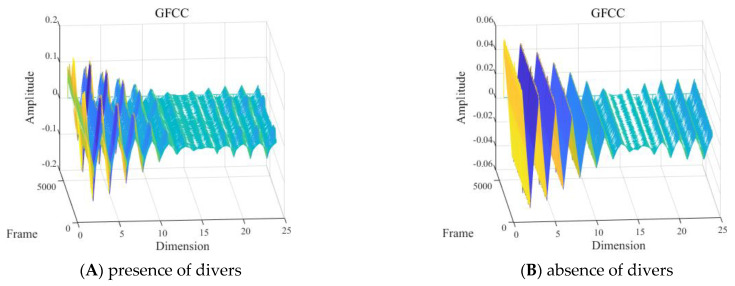
Gammatone frequency cepstral coefficient (GFCC) feature with the: (**A**) presence or (**B**) absence of divers.

With other conditions being equal, the GFCC feature of signals with and without divers underwater within the 2 to 8 kHz frequency band is shown in Figure 14.

As shown in Figure 14, within the frequency range of 2 to 8 kHz, the presence or absence of divers significantly affected the intuitive characteristics of GFCC features. The GFCC features with divers presented obvious peaks and valleys in the time domain, while the GFCC features without divers tended to be flat in the time domain. The amplitude of the time−domain peak of the GFCC features with divers was approximately three times that of the GFCC features without divers.

(2) Different numbers of divers underwater

**Figure 15:**
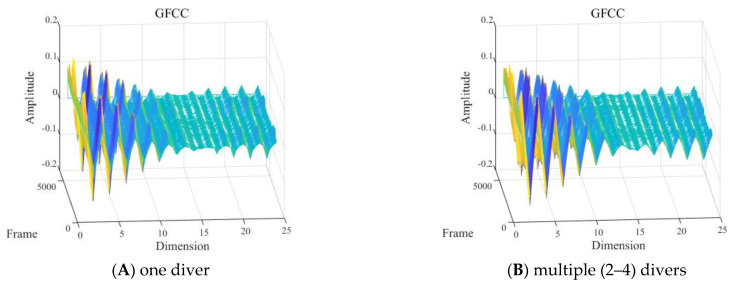
Gammatone frequency cepstral coefficient (GFCC) feature with the presence of: (**A**) one or (**B**) two to four divers.

With other conditions being equal, the GFCC feature of the signals of one diver and multiple (2–4) divers underwater in the 2 to 8 kHz frequency band is shown in Figure 15.

As shown in Figure 15, it was difficult to visually determine the influence of different numbers of divers on the GFCC features of the signal in the frequency band of 2 to 8 kHz.

(3) Different SNRs

**Figure 16:**
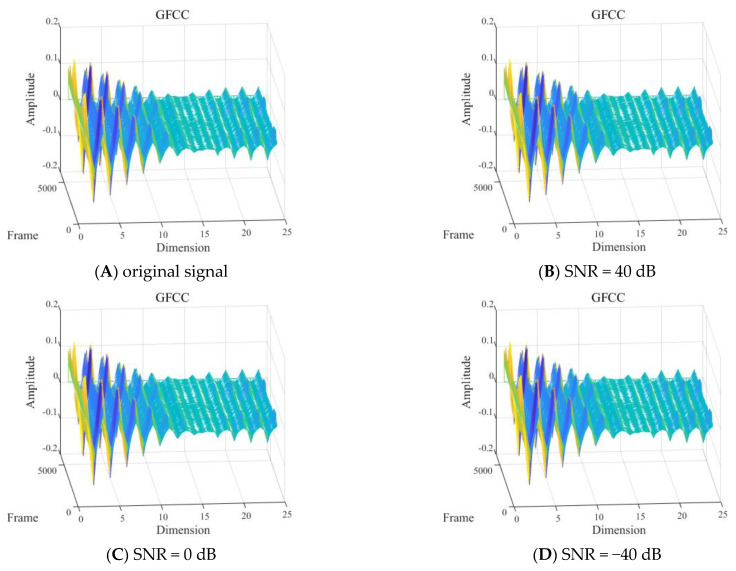
Gammatone frequency cepstral coefficient (GFCC) feature of one diver with: (**A**) the original signal−to−noise ratio (SNR), (**B**) 40 dB SNR, (**C**) 0 dB SNR, and (**D**) −40 dB SNR.

With other conditions being equal, the shallow sea background noise was added to the signals of one diver underwater in the 2 to 8 kHz frequency band to construct GFCC feature distributions for different SNRs, as shown in Figure 16.

As shown in Figure 16, it was difficult to visually determine the influence of different SNRs of the signal in the frequency band of 2 to 8 kHz on the MFCC features.

(4) Different detection distances

**Figure 17:**
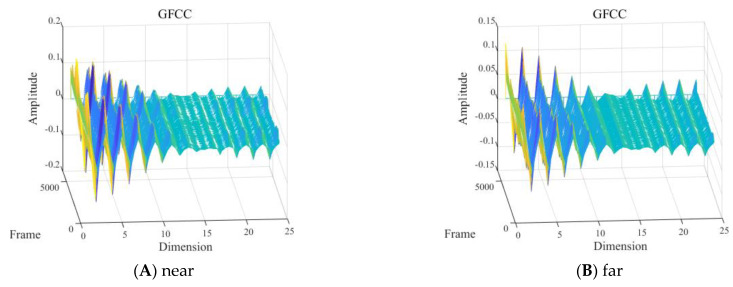
Gammatone frequency cepstral coefficient (GFCC) feature of one diver: (**A**) ~2 m and (**B**) ~20 m from the hydrophone.

With other conditions being equal, the GFCC features of the signals with one diver underwater in the frequency band of 2 to 8 kHz, near the hydrophone (about 2 m away) and far away (about 20 m away), are shown in Figure 17.

As shown in Figure 17, the impact of different detection distances on the intuitive characteristics of the diver’s signal GFCC was pronounced within the 2 to 8 kHz frequency band. The GFCC features of the diver signal near the hydrophone showed more distinct peaks and valleys in the time domain than those of the more distant detection distance, and the peak amplitudes of the two were basically the same.

### 3.4. Recognition Effect of an SVM Algorithm on the Three Types of Features

The above three types of features from 562 diver−related underwater acoustic signals under various operating conditions were extracted to an SVM in this work, including 424 signals from divers in pools, lakes, and shallow seas, as well as 138 signals without divers, e.g., background noise in these same water bodies, periodic motion noise from bionic robots in pools, and impact noise from throwing stones into the surface of a lake. The original signal duration of each data point was 10 sec, and the sampling rate was 90 kHz.

#### 3.4.1. Evaluation Indicators for Recognition Performance

In this study, each of the three types of features was divided randomly, with 50% as training data and the remaining 50% as test data, with no overlap between the training and test data. Result “1” indicated identification as “with divers”, and result “−1” indicated identification as “without divers”. To evaluate the recognition effect, several parameters were introduced in this paper, whose meanings were as follows:

TP: true positive (the recognition and actual results indicated the “presence of divers”).

TN: true negative (the recognition and actual results indicated the “absence of divers”).

FP: false positive (the recognition result indicated the “presence of divers”, but the actual situation was “absence of divers”).

FN: false negative (the recognition result was “absence of divers,” but the actual result was “presence of divers”);

Acc: recognition accuracy, indicating the proportion of recognition results that were consistent with the actual situation, calculated using Equation (18):

Acc = (TP + TN)/(TP + TN + FP + FN).(18)

Sen: recognition sensitivity, indicating the accuracy rate of recognizing the “presence of divers”, calculated using Equation (19):

Sen = TP/(TP + FN).(19)

Spe: recognition specificity, indicating the accuracy rate of recognizing the “absence of divers”, calculated using Equation (20):

Spe = TN/(TN + FP).(20)

#### 3.4.2. Recognition Results

To study the recognition performance of the three types of signal features, including the features of FMSE, MFCC, and GFCC with an SVM, these three types of features with an SVM were extracted separately, under the premise of different SNRs and other conditions in this paper, to recognize the “presence/absence of divers underwater”, and examine the influence of feature types on recognition performance.

**Figure 18:**
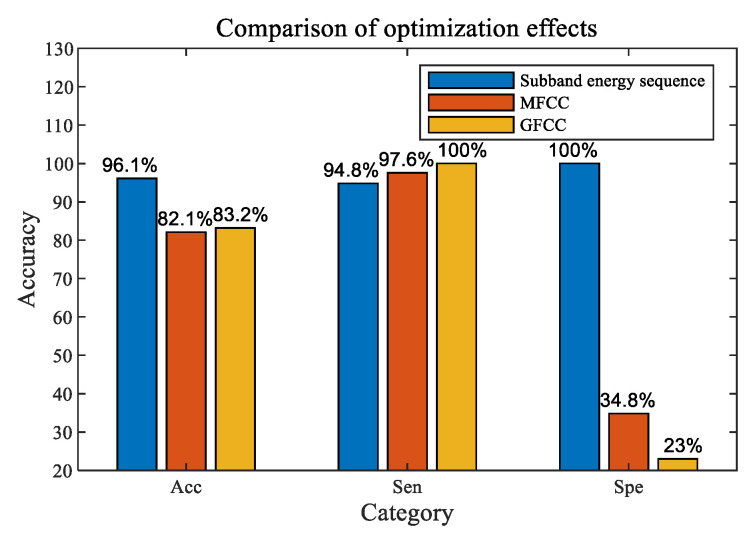
Comparison of three feature recognition effects. Note, MFCC, mel frequency cepstral Coefficient; GFCC, gammatone frequency cepstral coefficient; Acc, recognition accuracy, indicating the proportion of recognition results were consistent with the actual situation; Sen, recognition sensitivity, indicating the accuracy rate of recognizing the “presence of divers”; Spe, recognition specificity, indicating the accuracy rate of recognizing the “absence of divers”.

Based on the calculated results, the recognition performance of the three features with an SVM was compared (Figure 18).

The results in Figure 18 showed that: the recognition accuracy (Acc) of an SVM for the three types of features was 82% to 96%, the recognition sensitivity (Sen) was 94.8% to 100%, and the recognition specificity (Spe) varied greatly, indicating that the respective combination of these three types of features with an SVM all had a certain recognition ability for the presence and absence of divers underwater, while the FMSE method has the best overall performance.

## 4. Discussion

In summary, three types of features from the underwater acoustic signals of divers under various working conditions in pools, lakes, and shallow seas were extracted and combined with an SVM for recognition in this paper, as discussed below:

(1) The respective combinations of the three types of features extracted in this paper with an SVM all have a certain recognition ability for divers underwater. Among them, the FMSE features combined with an SVM have the best recognition performance. These three methods have good results in recognizing the “presence of divers” underwater, while the combination of MFCC and GFCC features with an SVM, respectively, has poor recognition performance for the “absence of divers” underwater.

(2) The recognition performance of the FMSE features of underwater acoustic signals from divers combined with an SVM is basically not affected by the signal SNR, training data volume, and so on. Its recognition accuracy can exceed 96%, recognition sensitivity can exceed 94%, and recognition specificity can reach 100%. The recognition probability for “presence of divers” and “absence of divers” is high, and the robustness is good, demonstrating the application potential of this method. The signal SNR has little effect on the recognition results of the FMSE features with an SVM. The main reason is that bandpass filtering was first performed in the 2 to 8 kHz frequency band during the formation of FMSE features, which plays a role in noise reduction filtering to some extent.

(3) The recognition accuracy and other performance of the combination of MFCC features of underwater acoustic signals from divers and an SVM will increase with the increase in the SNR and training data volume. The recognition accuracy of this method is stable at 81% to 91%. The recognition sensitivity is basically not affected by the SNR and always exceeds 94%, indicating a high probability and robustness of recognizing the “presence of divers”. However, the recognition specificity fluctuates greatly due to the influence of SNR and training data volume, ranging from 28% to 61%. This method has a generally low recognition probability for the “absence of divers”, which would limit the practical application of this method if it is not further optimized.

(4) Through the comparison of feature extraction results, it can be seen that the GFCC features distribution of underwater acoustic signals from divers is more prominent compared with MFCC features in the frequency band range of 2 to 8 kHz, and the periodic breathing characteristics of divers are very obvious in the time−domain. Moreover, GFCC features are more sensitive to the presence or absence of divers underwater and their distance from the hydrophone than MFCC features, and can more intuitively reflect the energy changes of the signals collected by the hydrophone.

(5) The recognition accuracy of the combination of GFCC features of underwater acoustic signals from divers and an SVM is less affected by the SNR, but it increases with the increase in training data volume. The recognition accuracy of this method is stable at 79% to 88%, and the recognition sensitivity exceeds 99%, which is higher than the other two methods, indicating a high accuracy and robustness of recognizing the “presence of divers”. However, the recognition specificity is always below 50%, and the overall recognition effect of this method for “absence of divers” is poor, which would limit the practical application of this method if it is not further optimized.

## 5. Conclusions

In this paper, the feature vectors obtained by the designed method of FMSE were extracted from underwater acoustic signal data of divers in various working environments, e.g., pools, lakes, and shallow seas. At the same time, the impacts of various conditions, such as different target numbers, SNRs, and detection distances, by this method were studied, which were compared with two other signal feature extraction methods: MFCC and GFCC. The research results indicate that the FMSE feature extraction method is robust and not affected by different target numbers, SNRs, or detection distances. After being combined with the recognition algorithm of SVM, the recognition accuracy of the three methods was 82% to 96% (FMSE was 96.1%, MFCC was 82.1%, and GFCC was 83.2%), the recognition sensitivity was 94.8% to 100% (FMSE was 94.8%, MFCC was 97.6%, and GFCC was 100%), and the recognition effectiveness varied greatly (FMSE was 100%, MFCC was 34.8%, GFCC was 23%), indicating the three types of features combined with SVM in this paper all have certain recognition capabilities for underwater targets of divers, while the FMSE method has the best overall performance. These three methods all had good results in recognizing the “presence of divers”, while the GFCC feature extraction method had the best performance, with a recognition sensitivity close to 100% and high robustness. The MFCC and GFCC methods had poor recognition performance for the “absence of divers”, with a recognition specificity of less than 60%, while the recognition method of FMSE feature extraction could achieve 100%. This indicates that the FMSE method is suitable for the underwater acoustic target recognition of divers.

However, the signal data used in this work still involved relatively few target types and operating conditions, and the analysis results were based on the fact that the SVM algorithm has not been optimized. In the future, data supplementation for multiple types of working conditions will be needed. If artificial intelligence recognition algorithms are introduced or the SVM algorithm is optimized by cross−validation optimization, particle swarm optimization, etc., the recognition effect of divers’ underwater targets will be further improved.

## Data Availability

The raw data supporting the conclusions of this article will be made available by the authors on request.
